# NMR Characterization of Angiogenin Variants and tRNA^Ala^ Products Impacting Aberrant Protein Oligomerization

**DOI:** 10.3390/ijms22031439

**Published:** 2021-02-01

**Authors:** Andrea Fagagnini, Miguel Garavís, Irene Gómez-Pinto, Sabrina Fasoli, Giovanni Gotte, Douglas V. Laurents

**Affiliations:** 1 Dipartimento di Neuroscienze, Biomedicina e Movimento, Sezione di Chimica Biologica, Università di Verona, Strada Le Grazie 8, I-37134 Verona, Italy; andrea.fagagnini@hotmail.it (A.F.); sabrina.fasoli@univr.it (S.F.); 2 Instituto de Química Física “Rocasolano”, Consejo Superior de Investigaciones Científicas, c/Serrano 119, E-28006 Madrid, Spain; mgaravis@gmail.com (M.G.); irene_gomez@iqfr.csic.es (I.G.-P.)

**Keywords:** protein oligomerization, angiogenin (h-ANG), tRNA, ALS, stress granules, NMR spectroscopy

## Abstract

Protein oligomerization is key to countless physiological processes, but also to abnormal amyloid conformations implicated in over 25 mortal human diseases. Human Angiogenin (h-ANG), a ribonuclease A family member, produces RNA fragments that regulate ribosome formation, the creation of new blood vessels and stress granule function. Too little h-ANG activity leads to abnormal protein oligomerization, resulting in Amyotrophic Lateral Sclerosis (ALS) or Parkinson’s disease. While a score of disease linked h-ANG mutants has been studied by X-ray diffraction, some elude crystallization. There is also a debate regarding the structure that RNA fragments adopt after cleavage by h-ANG. Here, to better understand the beginning of the process that leads to aberrant protein oligomerization, the solution secondary structure and residue-level dynamics of WT h-ANG and two mutants i.e., H13A and R121C, are characterized by multidimensional heteronuclear NMR spectroscopy under near-physiological conditions. All three variants are found to adopt well folded and highly rigid structures in the solution, although the elements of secondary structure are somewhat shorter than those observed in crystallography studies. R121C alters the environment of nearby residues only. By contrast, the mutation H13A affects local residues as well as nearby active site residues K40 and H114. The conformation characterization by CD and 1D ^1^H NMR spectroscopies of tRNA^Ala^ before and after h-ANG cleavage reveals a retention of the duplex structure and little or no G-quadruplex formation.

## 1. Introduction

Amyotrophic Lateral Sclerosis (ALS) is a neurodegenerative disease affecting motor neurons leading to spasticity, muscle atrophy, generalized weakness, paralysis and death [1]. The majority of the cases develop sporadically; however, about 10% of ALS patients bear genetic mutations. As a multifactorial disease, to date, at least 126 genes have been linked to ALS [2,3]. Among them, *TDP-43*, *SOD1*, *FUS/TLS*, *TARDBP*, *FIG4* and *C9ORF72* are some genes whose mutations are strongly implicated in the development of the pathology [4,5,6,7]. These genes affect key cellular processes, including RNA metabolism, stress response and autophagy [8]. In particular, oxidative stress can induce the formation of stress granules, i.e., membraneless organelles composed of certain RNAs and proteins. The formation of stress granules and the incorporation of mRNAs into these phase-separated microdroplets is mediated by the flexible, prion-like regions of heterogeneous ribonucleoprotein particle proteins [9]. Retention of mRNAs in stress granules arrests their translation, promoting health. However, excessive retention can lead to the formation of harmful oligomerization leading to amyloid-like aggregates by FUS and TDP-43 [10].

In addition to the abovementioned proteins, many genetic variants of human angiogenin (h-ANG) have also been reported in ALS patients [11]. h-ANG, also known as ribonuclease 5 (RNase 5) because it is included in the secretory pancreatic-type RNase super-family [12], is a 123 residue (14.1 kDa) protein [13] present in plasma [14], cerebrospinal and amniotic fluid [15] and in tumor microenvironments. Indeed, high h-ANG levels have been implicated in enhanced ribosome production and the formation of new blood vessels in cancer [16,17]. Despite being a pancreatic-like RNase, h-ANG is 10^5^–10^6^ less active against ssRNA than bovine pancreatic RNase A, the super-family prototype. Nevertheless, its ribonucleolytic activities on tRNA and rRNA are essential to its roles in preventing ALS and promoting cancer [18,19,20]. As revealed by X-ray crystallography, h-ANG’s structure is very similar to that of RNase A except that h-ANG lacks RNase A’s short disulfide bond (C65–C72) and has an additional short 3_10_ helix (residues 118–121), right at the C-terminus, which is not present in the prototype. In h-ANG, Q117′s side chain partly blocks the active site and accounts for part of the decreased activity of h-ANG with respect to RNase A [21], and Ile119 and Phe120 have been reported to make hydrophobic contacts that maintain Q117 in position [22].

**Figure 1:**
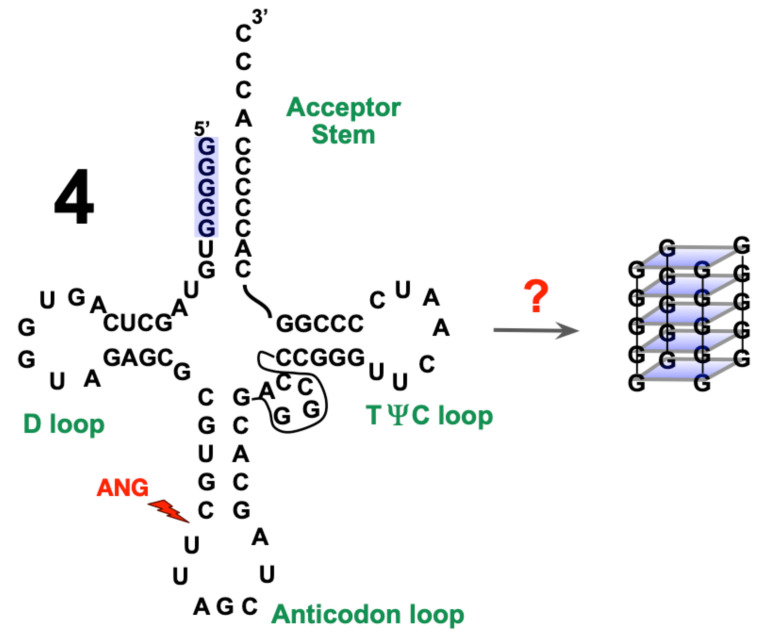
In cells, h-ANG is normally inhibited by the Ribonuclease Inhibitor (RI), which is highly sensitive to denaturation under mild oxidizing conditions [26]. Under cell stress conditions, RI could unfold, freeing h-ANG, which then cleaves tRNA in the anticodon loop (red lightning bolt) [27]. In this scenario, the 5′ tRNA fragment, called tiRNA, has been shown to induce the formation of stress granules. The absence of this h-ANG activity is linked genetically to Amyotrophic Lateral Sclerosis (ALS). Four tiRNA have been proposed to tetramerize to form a G-quadruplex structure, although high resolution corroboration of this point is still necessary.

It was demonstrated that, under stress conditions, h-ANG is able to promote the cleavage of human tRNA^Ala^, generating a 5′-tiRNA^Ala^ fragment endowed with a protective function that prevents harmful protein oligomerization in cell stress response [20,23]. The mechanism(s) through which h-ANG could promote the survival of motor neuron is (are) still unknown, but it has been hypothesized that the cleavage of tRNA^Ala^ allows the produced 5′-tiRNA^Ala^ fragment to adopt a G-quadruplex conformation, which is responsible for promoting stress granule formation, causing the inhibition of protein synthesis and favoring the motor neuron survival [24,25] (Figure 1). However, the proposed formation of a G-quadruplex structure was based only on a dye-binding experiment and other indirect assays [25].

In recent years, the enzymatic activity and 3D structure of many h-ANG variants linked to ALS have been characterized by Acharya’s laboratory using X-ray crystallography [28,29]. However, a few interesting variants did not crystallize and the R121C mutant was covalently modified by adduct formation with glutathione. Furthermore, the UV spectroscopy-based enzymatic assay on commercial yeast tRNA did not reveal the position of the cleavage or whether the cleaved RNA remained intact as a duplex, unfolded or transformed into a G-quadruplex. In principle, the conformation of these h-ANG variants and the tRNA cleavage product could be addressed by NMR and CD spectroscopies. Over the last two decades, the conformation in solution of h-ANG has been characterized by previous NMR studies using ^15^N [30] or ^13^C,^15^N labeled samples [31]. These studies have confirmed that the structure in the solution is similar to that found in the crystal, although there are some conflicting results regarding whether or not the small C-terminal 3_10_ helix (residues 117–121) is present. In addition, the solution dynamics of the protein have not been characterized.

To address these issues, we have characterized the solution conformation of WT h-ANG using multidimensional NMR spectroscopy and obtained the secondary structure by measuring ^13^C and ^1^Hα chemical shifts as well as ^1^HN-^1^Hα coupling constants. We assess h-ANG’s solution dynamics by {^1^H}-^15^N relaxation measurements and the protection against H/D exchange. These measurements provide additional information regarding the presence of helical structure at the C-terminus. NMR spectroscopy has also been used to characterize three variants (H13A, C39W and R121C) at positions reported to be genetically linked to ALS [11,32,33]. Mutation of the catalytic H13 very strongly reduces the enzymatic activity [29], as well as the angiogenesis induction [34] and might also affect the potential capacity of h-ANG to self-associate through 3D domain swapping [35], as this residue forms key intersubunit contacts present in all RNase A domain-swapped oligomers [36,37,38].

The R121C variant is particularly interesting as it has been reported to be hyperactive relative to WT h-ANG [29]. This is puzzling considering that, based on the current working hypothesis, one would expect that more h-ANG activity would produce tiRNAs that protect against pathological protein oligomerization and/or massive aggregation. Finally, the cleavage of human tRNA^Ala^ by WT h-ANG or by the mentioned variants has been characterized by mass spectrometry and by CD and NMR spectroscopies in order to reveal the cleavage position and to assess possible structural changes in this substrate.

## 2. Results

### 2.1. Secondary Structure in WT h-ANG

**Figure 2:**
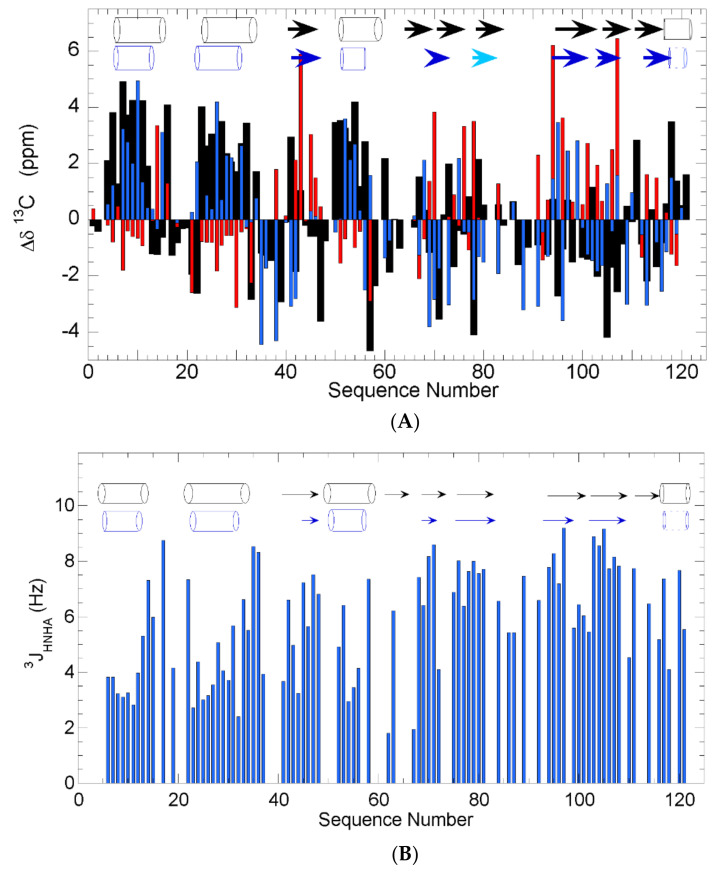
(**A**). Secondary structure from Chemical Shifts. The conformational chemical shifts of ^13^Cα (black bars), ^13^Cβ (red bars) and ^13^CO (blue bars) are shown. Based on the CSI rules [39], segments of secondary structure are identified and shown in blue as cylinders for helices and arrows for β-strands. The β-strand spanning residues 78–83 and the C-terminal helix, whose identification is doubtful, are shown in a lighter tone of blue and with broken lines, respectively. (**B**). ^3^J_NHHA_ coupling constants for WT h-ANG at 35 °C in pH 6.5 NaH_2_PO_4_/Na_2_HPO_4_ (NaPi) buffer (blue bars). The segments defined as secondary structural elements by X-ray crystallography (PDB 1ANG) and ^3^J_NHHA_ couplings are shown in black and blue, respectively, as cylinders for helices and arrows for β-strands. The short 3_10_ helix (blue dotted lines) is doubtful, as only residues 118 and 121 show low ^3^J_NHHA_ couplings.

We first characterized wild type (WT) h-ANG to provide a reference to determine the effects of the variants. Although the near physiological conditions of pH (6.5) and temperature (35 °C) are less favorable for NMR spectroscopy, the chemical shift assignments are largely complete for the nuclei which are most important for determining the backbone conformation: ^13^Cα = 93%, ^13^Cβ = 73%, ^13^CO = 76%, ^15^N = 82%, ^1^HN = 87%, ^1^Hα = 70%. The assignments have been deposited in the BMRB database under access code: 50,650 and the assigned ^1^H-^15^N HSQC spectrum is shown in Supplementary Figure S1. The analysis of the ^13^Cα, ^13^Cβ and ^13^CO chemical shifts confirmed that WT h-ANG is well folded with 5 β-strands and 3 α-helices, with the possible presence of a sixth β-strand and a C-terminal helix. (Figure 2A). Whereas these results are in general agreement with those observed previously by X-ray crystallography, the elements of secondary structure detected by NMR are generally somewhat shorter, and one β-strand is missing. Therefore, we sought to corroborate these results by a different approach.

Coupling constants provide an alternative way to identify a secondary structure as contiguous stretches of residues with low and high ^3^J_HNHA_ constants delineate helices and β-strands, respectively. According to these criteria, three α-helices and five β-strands are detected, which are generally somewhat shorter but otherwise in general agreement with previous results from X-ray crystallography (Figure 2B). The observation of i, i+1 NOE signals between ^1^HN I119–^1^HN F120, ^1^HN F120–^1^HN D121 and ^1^HN F120–^1^HN R121 are consistent with but do not prove the presence of helices. The somewhat shorter nature of these elements observed here, and the chemical shift results mentioned above, may be attributed to the lack of information for some unassigned residues as well as to the eight proline residues, which lack ^1^HN, or even to the more destabilizing nature of the conditions used here (35 °C, pH 6.5) relative to the crystallography ones.

### 2.2. Dynamics of WT h-ANG

**Figure 3:**
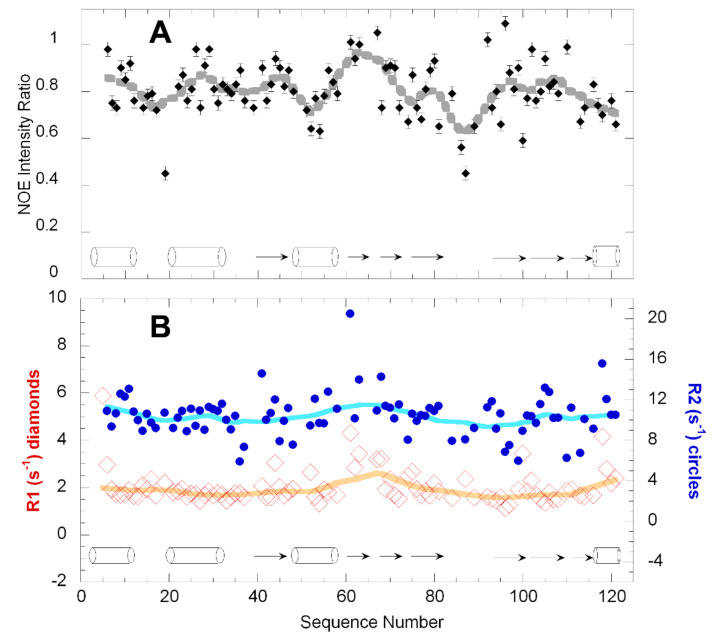
The secondary structure (from the X-ray crystal structure PDB 1ANG) is represented by cylinders for helices and arrows for β-strands. The thick lines represent the weighted average over the nearest measured values along the sequence. (**A**) Fast timescale dynamics assessed by the {^1^H}-^15^N NOE. (**B**) μs-ms timescale dynamics gauged by R_1_ (red open diamonds, left *y*-axis) and R_2_ (blue circles, right *y*-axis) relaxation rates.

The results of the ^15^N relaxation experiments are summarized in Figure 3. Overall, the high ratio of the {^1^H}-^15^N NOE shows that most of the secondary structural elements are almost fully rigid on ps/ns timescales (Figure 3A), while a modest flexibility is observed in the loops as well as the C-terminal α-helix.

Based on the average R_1_ and R_2_ values for residues in secondary structural elements (Figure 3B), a correlation time of 5.1 ns can be estimated. Based on an empirical relation, this correlation time corresponds approximately to a solution molecular weight of 9 kDa. As this value is not larger than the molecular weight of 14 kDa calculated from the sequence, it strongly suggests that the protein is monomeric under these conditions.

**Figure 4:**
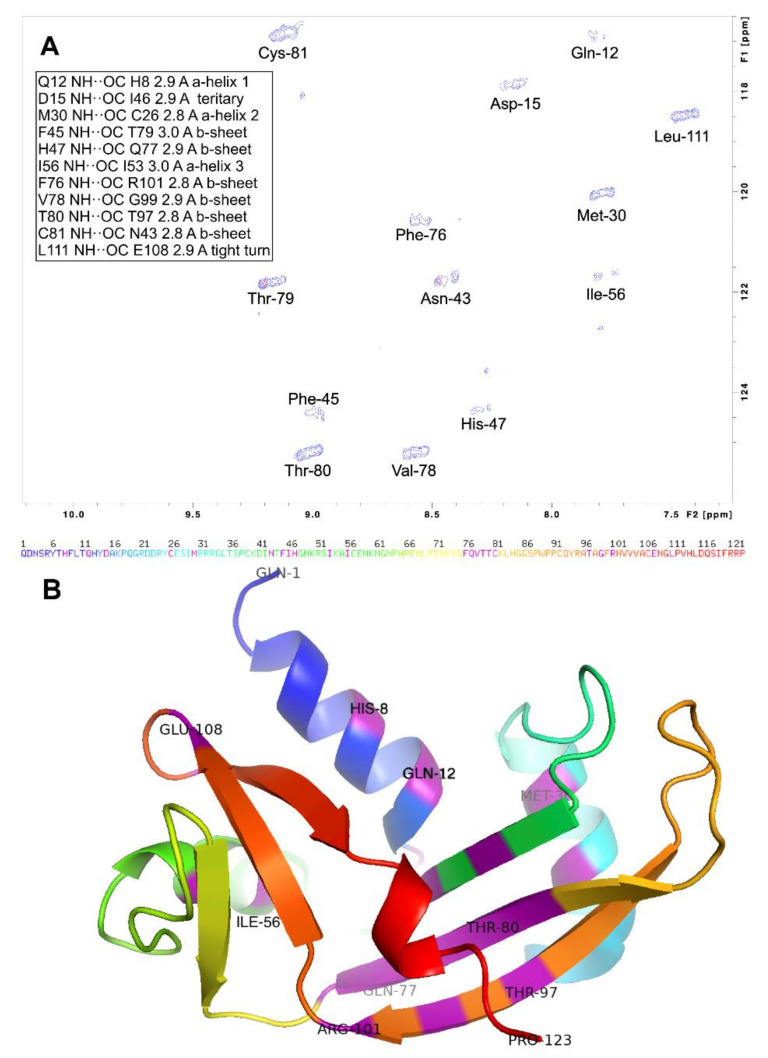
H/D exchange reveals protected amide H in h-ANG. (**A**). ^1^H-^15^N spectrum of h-ANG exchanging in D_2_O at pH* 6.5, 35 °C. Blue contours, first spectrum, recorded one hour after the start of exchange. Red contours (only visible for T79 and N43) represent the last spectrum recorded one day after the start of exchange. The inserted table lists the putative acceptors and donors of the H-bonds (as seen in PDB 1ANG) that protect these HN against exchange, the distance between N and O (in Å) and the structural element where they are located. (**B**). The sequence (above in the one-letter code) and X-ray crystal structure of h-ANG (PDB 1ANG) shown as a ribbon diagram colored blue (N-terminus) to red (C-terminus). Residues which participate either as donors or acceptors in H-bonds that afford protection against H/D exchange are colored purple. In addition, some of these residues and the N- and C-terminal residues are labeled.

Finally, the residue-level conformational stability of h-ANG was probed by H/D exchange (Figure 4A). Protected residues are located in all the protein’s secondary structure elements, except the C-terminal 3_10_ helix (Figure 4B). The β-strand composed of residues Q78-H84 is particularly rich in protected HN groups, implying a high local stability and rigidity on slow timescales.

### 2.3. Structural Changes and Dynamics of h-Ang Variants

**Figure 5:**
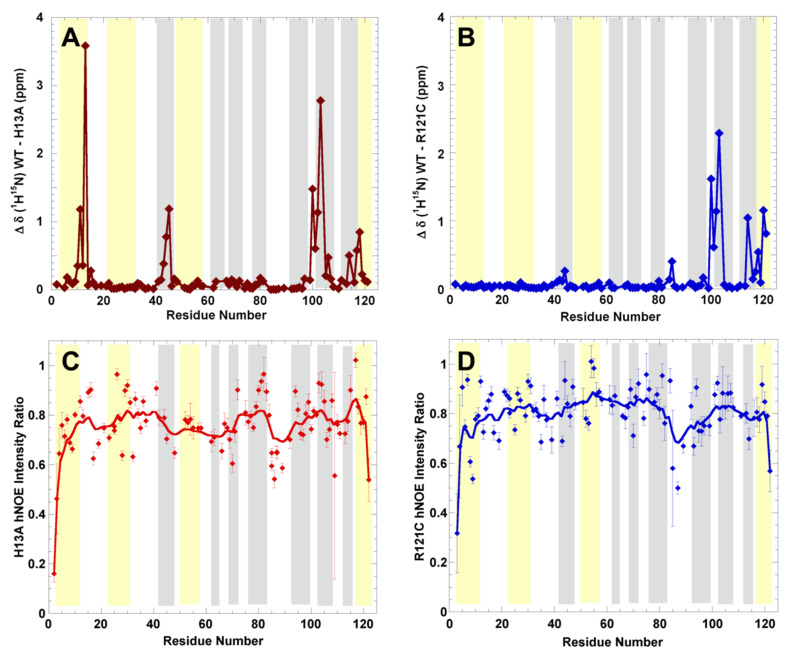
Characterization of the solution structure and dynamic of h-ANG variants H13A and R121C. (**A**). ^1^H-^15^N chemical shift perturbation in h-ANG variants H13A (red) and (**B**). R121C (blue) relative to WT reveal structural perturbations around the active site for H13A and more localized disruptions in the case of R121C. (**C**). ps-ns dynamics of variants H13A (red) and (**D**). R121C (blue) as assessed by the {^1^H}-^15^N NOE. In all panels, the helical regions (according to PDB 1ANG) are shaded yellow and β-strands are shaded gray.

The ^1^H-^15^N NMR spectra of the variants H13A and R121C were assigned and are shown in Supplementary Figures S2 and S3, respectively. The experimental chemical shifts are exquisitely sensitive to conformation. Overall, the conservation of most of them indicates that the 3D structures of the variants are largely unchanged (Figure 5A). However, the H13A mutation does induce significant changes at sites which are well separated along the sequence and correspond to residues that form parts of the active site (K40 and H114), as well as at residues 100–103. Indeed, the mutation of H13 is expected to weaken the phosphate ion binding. Whereas this binding was reported to have little effect on the active site conformation [40], it could significantly alter the magnetic environment. Therefore, it is possible that the changes seen are due to structural alterations caused directly by the H13A substitution, or changes in the magnetic environment due to a reduced presence of phosphate ligand, or both. In contrast to the alterations seen for H13A, the ones induced by R121C are localized to nearby residues along the sequence, but like H13A, changes are seen in residues 100–103. Composing the turn connecting the third and second to last β-strands, residues 100–103 are fairly close to the 3_10_ helix in the PDB 1ANG X-ray structure [41]. Although the C39W variant was not assigned, its ^1^H-^15^N HSQC (Supplementary Figure S4) strongly resembles those of the other variants and WT h-ANG, which firmly suggests that this mutant is also well folded.

### 2.4. Assessment of tRNA^Ala^ Structure Following Cleavage by h-ANG

**Figure 6:**
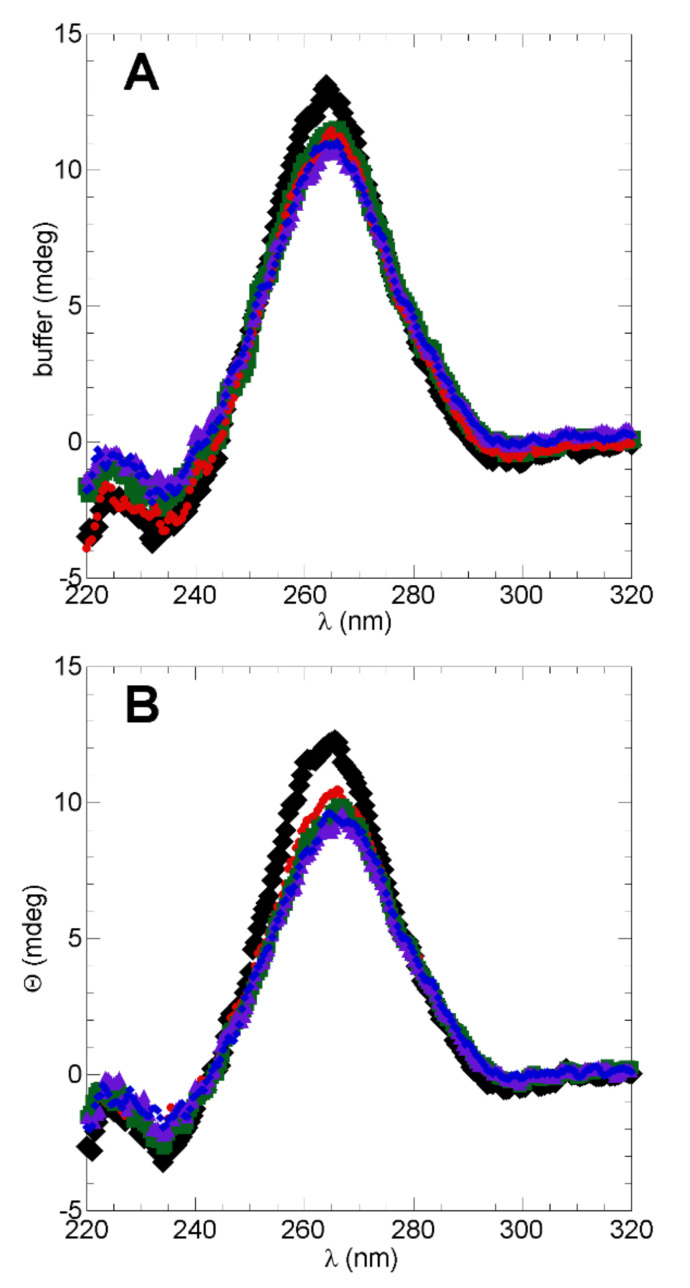
UV CD Spectra of tRNA^Ala^ before and after h-ANG Cleavage. CD spectra of 20 μM tRNA^Ala^ in 100 mM KCl, 1.0 mM MgCl_2_, 20 mM NaPi, pH 6.5, 25 °C. Black = tRNA^Ala^ in buffer; Green = tRNA^Ala^ + wt h-ANG, Red = tRNA^Ala^ + H13A h-ANG, Purple = tRNA^Ala^ + W39C h-ANG, Blue = tRNA^Ala^ + R121A h-ANG. (**A**). Spectra right after incubation for cleavage. (**B**). Spectra following cleavage and after applying a heating followed by a slow cooling step for possible structural annealing.

The CD spectra of tRNA^Ala^ alone, or following cleavage exerted by h-ANG or its variants are shown in Figure 6A. The observation of a strong maximum near 265 nm and a weak minimum near 235 nm for tRNA^Ala^ alone are characteristics of A-type double helix and are consistent with the presence of the native, folded structure [42]. A modest decrease in the intensity of these peaks is evident when the tRNA^Ala^ was incubated in the presence of WT h-ANG or its variants, even in the H13A variant whose enzymatic activity is strongly diminished. Since the expected cleavage in the anti-codon loop of tRNA^Ala^ (Figure 1) was confirmed by mass spectroscopy for the R121C variant, we wondered whether it is possible that tRNA^Ala^ retained its A-form duplex structure following its cleavage. In this scenario, the cleaved tRNA^Ala^ would be trapped in an energetic minimum; namely, the A-form duplex, which delays the formation of the putative quadruplex structure. To test this point, we subjected the samples to a heating plus slow cooling “annealing,” which should separate the duplex and allow the 5′tiRNA fragments to tetramerize to form a G-quadruplex. Nevertheless, following this annealing step, the CD spectra showed only small variations (Figure 6B). This suggests that most of the tRNA^Ala^ fragments may retain A-helix-like structures following cleavage. Nevertheless, it is important to bear in mind that parallel G-quadruplexes, i.e., those with their four strands running in the same orientation have a CD spectrum that is very similar to the A-form duplex CD spectra [42,43]. This may account for the absence of substantial change(s) in the CD spectra following the annealing.

**Figure 7:**
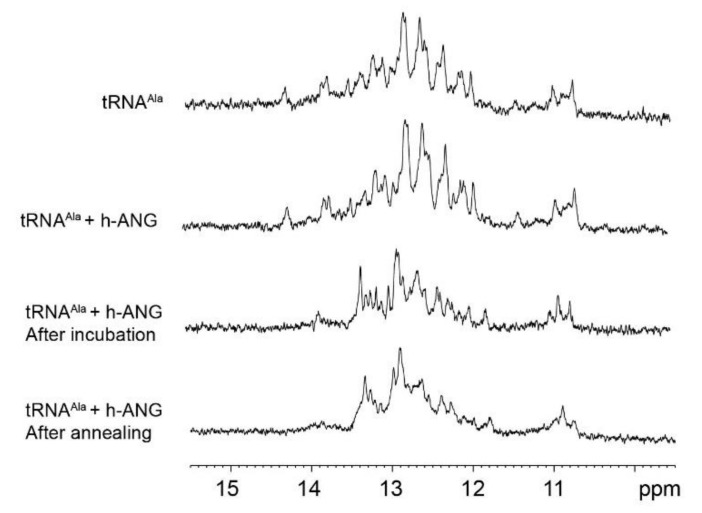
1D ^1^H Spectra of tRNA^Ala^ (imino HN region) before and after h-ANG cleavage. The top spectrum corresponds to tRNA^Ala^ in KPi buffer. The second spectrum from the top shows the imino region upon addition of ^1^/_20_ molar equivalent of h-ANG protein. The next spectrum (third from the top) corresponds to the previous RNA:h-ANG mixture after ON incubation. The bottom spectrum belongs to the mixture after being subjected to annealing at 90 °C for 10 min and slow cooling down to room temperature.

To further test this point, we employed NMR spectroscopy, which can distinguish the ^1^H nucleic acid imino signals of G-quadruplex (10.5–12.0 ppm) versus duplex (12.0–14.0 ppm) structures [44,45]. NMR spectra of tRNA^Ala^ alone in 25 mM K_2_HPO_4_/KH_2_PO_4_ (KPi) buffer, upon incubation with a 1/20th equivalent of h-ANG and finally after subjecting this mixture to an “annealing” step, are shown in Figure 7. The initial spectrum shows many signals in the chemical shift range of 12.5–14.2 ppm, which corresponds to imino protons participating in canonical G:C and A:U base pairs. In addition, a few signals are detected between 10 and 12 ppm which are characteristic of imino protons stabilizing G-tetrads. Nevertheless, in the case of tRNA, the latter signals have been observed previously and were attributed to the effect of modified bases (which are absent here) or tertiary hydrogen bonding between the D-loop and the TΨC loop [46,47]. Just after the addition of ^1^/_20_ molar equivalent of h-ANG, no significant changes are detected in the imino region. Following overnight (ON) incubation at 37 °C; however, several signals in the spectral region characteristic of duplex structure have shifted or disappeared, whereas many others remain unchanged. Notably, just slight changes are observed even after an annealing step in the imino region characteristic of G-quadruplex (Figure 7).

## 3. Discussion

### 3.1. Secondary Structure of h-ANG under Near-Physiological Conditions of pH and Temperature

The characterization of the conformation of h-ANG in solution by two independent criteria, namely, chemical shifts and the ^1^HN-^1^HA coupling constants, under near physiological conditions of pH and temperature reveal that the elements of secondary structure detected do seem to be moderately shorter than they are in X-ray crystal structures [41]. As another NMR study of h-ANG’s solution structure under conditions of somewhat lower pH (5.0) and temperature (25 °C) [31] also identified somewhat shorter elements of secondary structure than in the crystal structures, this suggests that the more stabilizing environment of the crystal lattice and conditions may modestly increase the length of secondary structure elements. On the other hand, an earlier NMR study by Lequin et al. [30] performed at pH 5.0 and 30 °C used ^1^Hα (but no carbon) conformational chemical shifts, ^3^J_HNHA_ couplings, H/D exchange and NOE criteria to define secondary structure and in general found secondary structure elements similar in length, save the first α-helix, to those observed by crystal studies. In the Lequin et al. study [30], NOEs were key for identifying the fourth, C-terminal helix (composed of residues 117–121), and it is worth pointing out that NOE correlations are less quantitative and can arise from partly populated structures. Considering all these results together, it is likely that the secondary structural elements are slightly shorter in solution conditions and that the C-terminal helix is unraveled part of the time. This may be of consequence to the important role played by Q117 [41] or by a homologous E117 in bovine h-ANG [48] in blocking the active site and strongly downregulating angiogenin’s activity.

### 3.2. h-ANG Adopts a Highly Rigid Structure in Solution

In this first characterization of the h-ANG dynamics by ^15^N relaxation, the protein was found to be highly rigid on ns/ps timescales. The more flexible regions, namely the N- and C-termini and the loop composed of residues 85–90, are also flexible in human RNase 1 [49] and show relatively high RMSD values in the h-ANG solution structure [30]. The protected residues are a subset of those observed by Lequin et al. [30]. This is reasonable considering that their experiment was conducted at pH 5.0 and 25 °C where H/D exchange is 80 times slower. In a broader sense, the positions showing slow exchange are well conserved throughout the RNase A super-family, and are even similar in the crystalline state [50]. Under the conditions studied here, the most protected residues in h-ANG exchange some 10^5^ times slower than an unprotected peptide. Though impressive, this level of protection is lower than that measured for homologous ribonucleases such as RNase A [51] or RNase 3, i.e., the Eosinophil Cationic Protein [52], which must function under tough extracellular conditions. As conformational stability can affect protein turnover and activity [53], the lower stability of h-ANG may be one way its activity is regulated. Future experiments would be required to test whether this lower stability is due to h-ANG having one less disulfide bond than the other super-family RNases, or to W89, a large aromatic residue which is constrained in a hyper-exposed position by two neighboring proline residues [41,54].

Regarding the ALS-linked variants H13A and R121C [33], our results have revealed that they all share a similar solution structure and are highly rigid on ps/ns timescales. This means that their implication to ALS is probably not due to destabilization and amyloid formation, as has been instead proposed for destabilized Superoxide Dismutase variants [55]. Conversely, it could be related to the production of RNA fragments which promote a healthy stress granule response.

### 3.3. On the Conformation of tRNA^Ala^

The CD and NMR spectroscopic results obtained here evince that most of the tRNA^Ala^ fragments produced by h-ANG ribonucleolytic activity may not actually adopt G-quadruplex structures as has been previously proposed [24,25]. This result is not surprising, considering that G-quadruplexes may have a conformational stability lower than duplex structures under some conditions [44]. Moreover, it is important to point out that the possible G-quadruplex structure formed by tiRNA^Ala^ requires four G-rich RNA fragments. Thus, the RNA concentration is an important factor influencing the formation of this tetrameric G-quadruplex. Due to technical limitations, our experiments were conducted under relatively low concentrations of tRNA^Ala^ and K^+^. Additionally, we cannot rule out that G-quadruplex formation may take place in the presence of components mimicking the cellular environment, such as crowding agents or protein binders like Y-box binding protein 1 [25]. Since higher concentrations of these substances are found in cells and will tend to favor G-quadruplex stability, future experiments are required to fully evaluate the possible physiological importance of the G-quadruplex conformation of tiRNA. Moreover, we cannot formally rule out that part of the cleaved tRNA^Ala^ may adopt a G-quadruplex while the rest of the molecule continues forming duplex. The changes observed in the region between 10 and 12 ppm of the NMR spectra upon incubating the tRNA with h-ANG may correspond to the presence of a less abundant population of a G-quadruplex formed by G-rich tiRNA fragments. In fact, a very similar tiRNA has been reported to form multimeric G-quadruplexes which coexist in equilibrium with other species [25]. In the coming years, the application of 2D heteronuclear NMR spectroscopic methods, which have shown recent success in resolving nucleic acids with mixed duplex/G-quadruplex structures [56], could resolve this issue.

### 3.4. Conclusions and Implications for Protein Oligomerization

Protein oligomerization is crucial for a myriad of physiological and pathological biochemical events. Harmful protein oligomerization has been observed to occur in stress granules by TDP-43 [57] and FUS [58]. These oligomers, which evolve into amyloid-like conformations, have been reported to harm cells by removing vital activities, by being toxic, or both [59,60]. h-ANG cleaves certain tRNAs to produce fragments that favor a healthy stress response, and based on the results reported here, we conclude that mutations at disease-linked residue positions do not substantially affect protein structure or rigidity. These findings, as well as previous enzymatic assays and crystallography results [11,29,41,48,61,62], provide strong evidence that these mutations act by affecting ribonucleolytic activity or by inferring with the cell surface receptor binding site. Future experiments are needed to further clarify the pathological mechanism(s) of these mutations. Our conformational characterization of the cleaved tRNA^Ala^ did not provide conclusive evidence for a G-quadruplex structure; instead, they are more consistent with the retention of some duplex structure under these conditions. Therefore, the conformations adopted by cleaved tRNA, and how the conformation induces a beneficial stress response, are still unclear. Future investigations to monitor protein oligomerization in reconstituted stress granules should help advance our understanding.

## 4. Materials and Methods

### 4.1. ANG Mutants Cloning

The plasmid pcDNA-ANG carrying the gene for WT ANG was already available in our laboratory. To produce h-ANG mutants, the following primers were used:

- H13A, F: 5′-CATTTCCTGACCCAGGCCTATGACGCTAAAC-3′,
- R: 5′-GTTTAGCGTCATAGGCCTGGGTCAGGAAATG-3′;
- C39W, F:5′ CCGTGGGTTAACTAGCCCGTGGAAAGATATC 3′,
- R: 5′ GATATCTTTCCACGGGCTAGTTAACCCACGG 3′;
- R121C, F: 5′-GTCCATCTAGATCAGTCTATCTTCTGCAGGCCT-3′,
- R: 5′-AGGCCTGCAGAAGATAGACTGATCTAGATGGAC-3′.

Site-directed mutagenesis was carried out through PCR, using QuikChangeII (Agilent, Santa Clara, CA, USA) mutation kit. The amplification products were sequenced in order to confirm the occurred mutations.

### 4.2. Expression and Purification of WT ANG and Mutants

BL21(DE3) cells containing pcDNA plasmids coding for WT ANG, H13A, C39W and R121C were incubated at 37 °C in 1 L of TB enriched with ampicillin until reaching the OD of 1.5. Cells were then harvested and resuspended in 500 mL of M9 medium enriched with ^13^C-glucose, ^15^NH_4_Cl and ampicillin, and hence, after 1 h incubation at 37 °C, they were induced ON at the same temperature, through the addition of 0.4 mM IPTG. Cell lysis and protein purification from inclusion bodies were performed according to the protocol by Notomista et al., developed for Onconase, another member in the RNase A super-family, although being of amphibian origin [63]. The protein samples were purified using a Superdex 75 10/300 HR column attached to an ÄKTA-FPLC system (GE Healthcare, Chicage, IL, USA), and equilibrated with a 50 mM NaPi buffer, pH 6.5.

### 4.3. Synthesis and Purification of tRNA^Ala^

tRNA^Ala^ was produced by T7 in vitro transcription using the following ssDNA sequence as a template: 5′-GGGTGGAGGTGCCGGGGATTGAACCCGGGGCCTCGTGCATGCTAAGCACGCGCTCTACCACTGAGCTACACCCCC-3′, (IDT, Coralville, IA, USA) containing the T7 RNA polymerase promoter sequence 5′-TATAGTGAGTCGTATTA-3′ on its 3′ end. After T7 primer hybridization, small scale transcription reactions (50 μL) were carried out in order to determine the concentration of MgCl_2_ yielding the highest amount of RNA. Then, the preparative reaction was carried out in a total volume of 10 mL, with a MgCl_2_ concentration of 30 mM. The reaction was stopped by adding 60 mM EDTA, and the resulting RNA was precipitated by addition of 300 mM (final concentration) sodium acetate pH 5.2 and 30 mL of 100% cold ethanol and ON incubation at −20 °C. The RNA precipitate was rinsed with 70% cold ethanol solution. tRNA^Ala^ was, hence, purified through 8% 7 M urea denaturing PAGE, and eluted from the gel by using the Elutrap device. The tRNA^Ala^ concentration was measured using a Nanodrop 2000 spectrophotometer.

### 4.4. NMR Experiments

Assignment and determination of secondary structure: ^13^C,^15^N-labeled h-ANG samples were prepared in 90% H_2_O/10% D_2_O in 50 mM NaPi buffer, pH 6.5, containing sodium 2,2-dimethyl-2-silapentane-5-sulfonate (DSS) as the internal chemical shift reference. At this concentration of NaPi and similar pH, a phosphate ion has been reported to occupy the active site and to be bound to Q12, H13, K40, H114 and L115 [40]. A series of 2D and 3D spectra, namely: ^1^H-^15^N HSQC, HNCO, HNHA, CBCANH, CBCA(CO)HN and HN(CA)CO, were recorded at 35 °C with a protein concentration ranging from 0.5 to 1 mM using a Bruker (Billerica, MA, USA) Avance 800 MHz NMR spectrometer, equipped with a triple axis resonance cryo-probe with Z-gradients. Manual peak assignment was performed using the program SPARKY [64]. The obtained chemical ^13^CA, ^13^CO and ^13^CB chemical shifts were compared to those predicted for a statistical coil, using the CSI approach [39], under these pH and temperature conditions [65] to identify elements of secondary structure. As an orthogonal approach to identify secondary structure elements, the ratio of the ^1^Hα-^1^HN crosspeak: ^1^HN-^1^HN diagonal peak intensities of a 3D HNHA spectra were determined and used to calculate the ^3^J_HNCHA_ coupling constants [66]. Finally, crosspeaks in a 3D ^1^H-^1^H-^15^N NOESY HSQC experiment, recorded with an 80 ms mixing time, were examined for the C-terminal residues to test for evidence of 3_10_ helix formation.

### 4.5. NMR Experiments: Ps/ns Timescale Dynamics through the {^1^H}-^15^N NOE

Fast timescale backbone dynamics were gauged by measurement of two ^1^H-^15^N HSQC type spectra with and without the application of the {^1^H}-^15^N NOE, which were recorded in an interleaved fashion. A long 10-s delay between pulses was employed to ensure complete recovery between pulses. No non-uniform sampling or linear prediction were used in the recording or processing of the spectra that were in turn integrated using Topspin 2.1 software. The experimental uncertainties were estimated from the standard deviation of the integrals of peakless regions of the spectrum.

### 4.6. NMR Experiments: μs/ms Timescale Dynamics via Longitudinal (R_1_) and Transverse (R_2_) Relaxation Rates

To explore the backbone dynamics of h-ANG on microsecond/millisecond timescales, we used the approach of Kay and co-workers [67] by recording eight spectra with delays of 20, 60, 240, 100, 860, 460, 150, 1500 and 2500 ms to determine R_1_ rates and seven spectra with delays of 8, 16, 36, 76, 120, 300 and 900 ms to determine R_1rho_. Then, R_2_ relaxation rates were calculated using the formula:

R_2_ = R_1rho_ − R_1_ (cos θ)^2^/(sin θ)^2^,

where θ = arctan (1700 Hz/δ ^15^N signal − δ ^15^N spectral center).

### 4.7. NMR Experiments: H/D Exchange

To assess the residue-level stability and slow dynamics, hydrogen/deuterium exchange was started by dissolved lyophilized ^13^C, ^15^N h-ANG in 100% D_2_O buffer, pH* 6.5, where pH* is the pH meter reading in D_2_O without being corrected for the deuterium isotope effect. Then, a series of 18 2D ^1^H-^15^N HSQC were recorded to monitor the exchange. Peaks were integrated using the Topspin 4.0.8 software (Bruker Biospin). Then, a single exponential decay function was fit to the peak integral versus time data to determine the observed rate constants for exchange. By comparing the observed rate constants to the values expected for a disordered polypeptide composed of the same sequence, the protection factors were calculated for this temperature and pH using the parameters reported by Bai and coworkers [68] and implemented on the Sphere HX webserver [69].

### 4.8. NMR Experiments: Assignment of h-ANG Variant NMR Spectra

3D HNCO, HNCA, CBCA(CO)NH and CBCANH spectra, as well as a series of 2D ^1^H-^15^N HSQC spectra filtered in ^13^C to select subsets of amino acid based on their side chains [70], were analyzed to assign the H13A and R121C variants.

### 4.9. Characterization of h-ANG Cleavage on tRNA^Ala^ Conformation

Circular dichroism (CD) was used to characterize the effect of h-ANG and its variants on tRNA^Ala^ structure. Samples of tRNA^Ala^ (20 μM) were incubated with buffer or WT h-ANG or variants (0.5 μM) in 100 mM KCl, 1.0 mM MgCl_2_ and 20 mM NaPi (pH 6.5) to afford cleavage. The integrity of the starting tRNA^Ala^ was confirmed by MALDI-TOF mass spectroscopy as a broad peak near the expected mass of 24,350 Da was observed. In the case of R121C, it was possible to confirm by MALDI-TOF mass spectroscopy that the tRNA^Ala^ was completely cleaved yielding fragments whose molecular weights of 15,115, 14,850 and 10,112 Da correspond approximately to the expected h-ANG cleavage site in tRNA^Ala^, which would produce fragments of 14,492 and 10,017 Da. CD spectra were recorded over a wavelength range of 220–320 nm, using a 50 nm/min scan speed and a 1.0 nm bandwidth. Four scans were averaged per spectrum, and the buffer spectrum was recorded and subtracted.

1D ^1^H NMR experiments with 0.10 mM tRNA^Ala^ were recorded at 37 °C on a Bruker 800 MHz (^1^H) spectrometer on samples dissolved in 25 mM KPi buffer at pH 6.5 containing 10% D_2_O (*v*/*v*), 1 mM MgCl_2_, and 0.5 mM DSS as the internal chemical shift standard. 1D ^1^H NMR spectra were registered using a 24-ppm sweep width and 2048 scans per spectrum on tRNA^Ala^ alone, tRNA^Ala^ + 0.05 mM h-ANG before and following ON incubation and annealing to assess the effect of ribonucleolytic cleavage on tRNA^Ala^ structure.

## Data Availability

The NMR chemical shift assignments for WT h-ANG have been deposited in the BMRB database under access code: **50650**.

## Supplementary Materials

The following are available online at https://www.mdpi.com/1422-0067/22/3/1439/s1, Figure S1: 2D ^1^H-^15^N HSQC spectrum of WT-h-Ang at pH 6.5, 35 °C; Figure S2: 2D ^1^H-^15^N HSQC spectrum of H13A h-Ang at pH 6.5, 35 °C; Figure S3: 2D ^1^H-^15^N HSQC spectrum of R121C h-Ang at pH 6.5, 35 °C; Figure S4: 2D ^1^H-^15^N HSQC spectrum of C39W h-Ang at pH 6.5, 35 °C.
