# Supplementary material for: NMR Characterization of Angiogenin Variants and tRNAAla Products Impacting Aberrant Protein Oligomerization
Source: Int J Mol Sci. 2021 Feb 1;22(3):1439. doi: 10.3390/ijms22031439 (PMC7867098; doi:10.3390/ijms22031439)

Supporting Material for:

NMR Characterization of Angiogenin Variants and $tRNA^{Ala}$ Products Impacting Aberrant Protein Oligomerization

by Andrea Fagagnini, Miguel Garavís, Irene Gómez-Pinto, Sabrina Fasoli, Giovanni Gotte and Douglas V. Laurents

Supplementary Figure S1: ^{1}H-^{15}N HSQC Spectrum of h-ANG (pH 6.5, 35 °C)

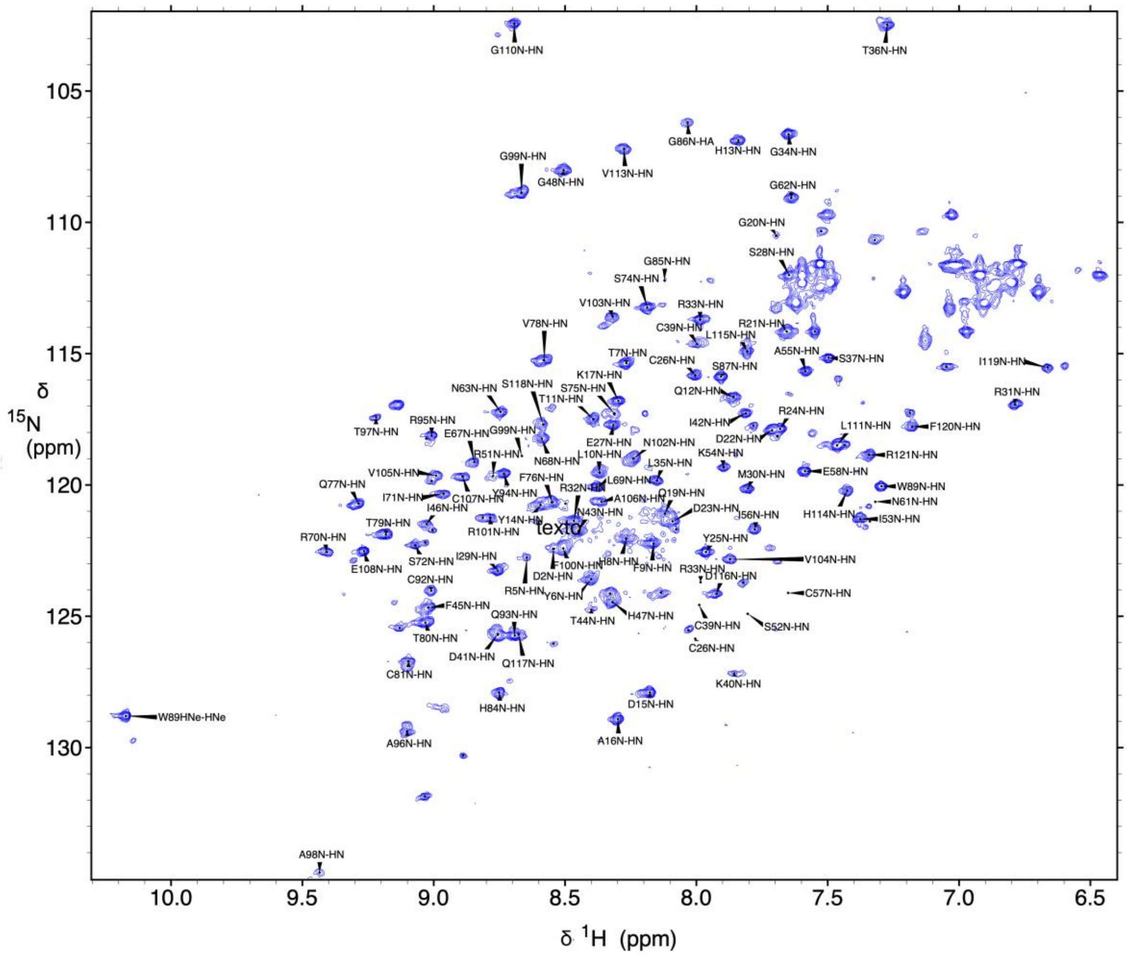

The ^{1}H-^{15}N-type spectrum of WT h-ANG with representative assignments.

Supplementary Figure S2: ^{1}H-^{15}N HSQC Spectra of H13A h-ANG (pH 6.5, 35 ℃)

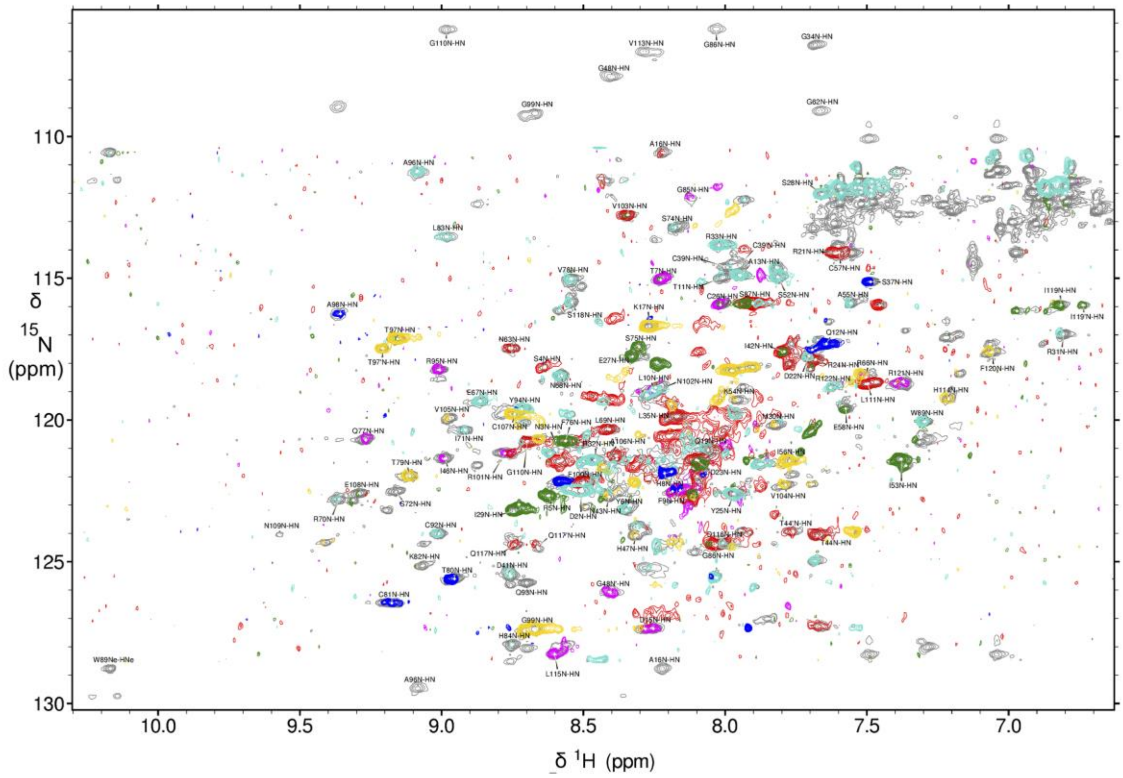

The ^{1}H-^{15}N-type spectrum of H13A h-ANG with representative assignments. For the central band of ^{15}N, additional ^{1}H-^{15}N-type spectra filtered in ^{13}C according to the ^{13}Cβ and ^{13}Cγ are shown in color. In these spectra, colors correspond to residues whose preceeding (*i-1*) residue are: A,V, I = **gold**, C,S= **green**, H,Y,F,W = **magenta**, G,N,D = **red**; T = **blue** and P,K,R,E,M,L,Q= **cyan**. Some doubled peaks are visible, *e.g.* T44, T97, Q117, I119. This behavior also appears in other variants and was previously reported by Lequin *et al.* under conditions of lower pH and temperature [25].

Supplementary Figure S3: 2D ^{1}H-^{15}N HSQC Spectrum of R121C h-ANG

(pH 6.5, 35 ℃)

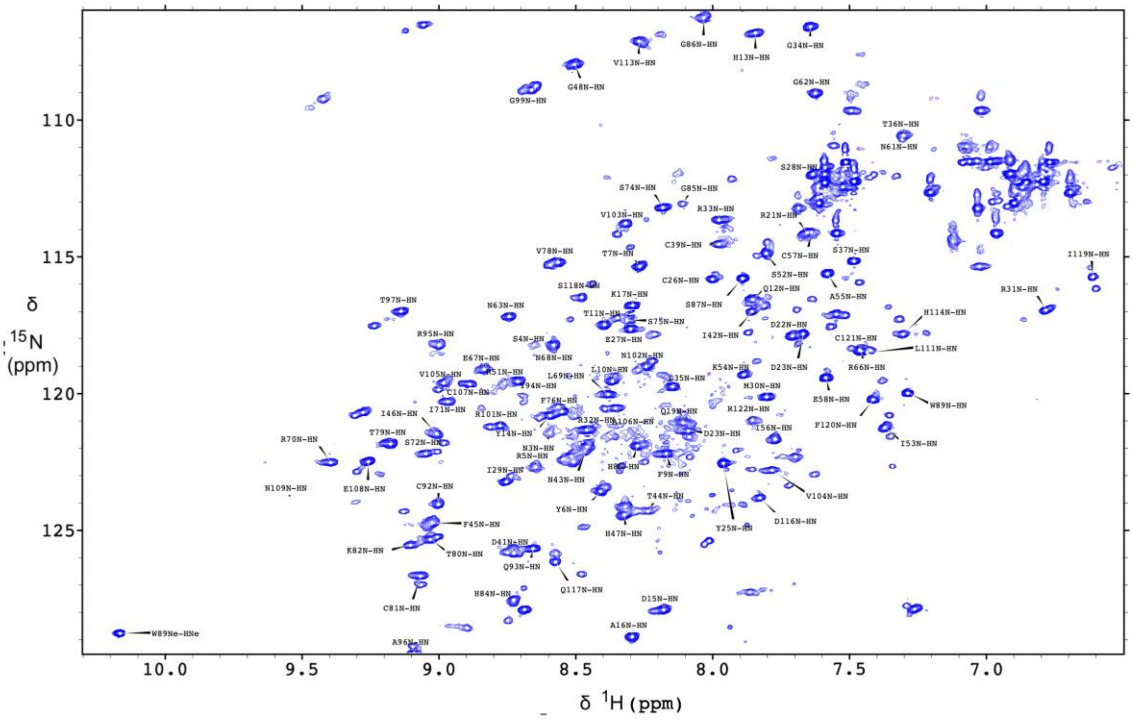

The ^{1}H-^{15}N-type spectrum of R121C h-ANG with representative assignments.

Supplementary Figure S4. 2D ^{1}H-^{15}N spectrum of C39W h-ANG recorded at pH 6.5, 35 ℃

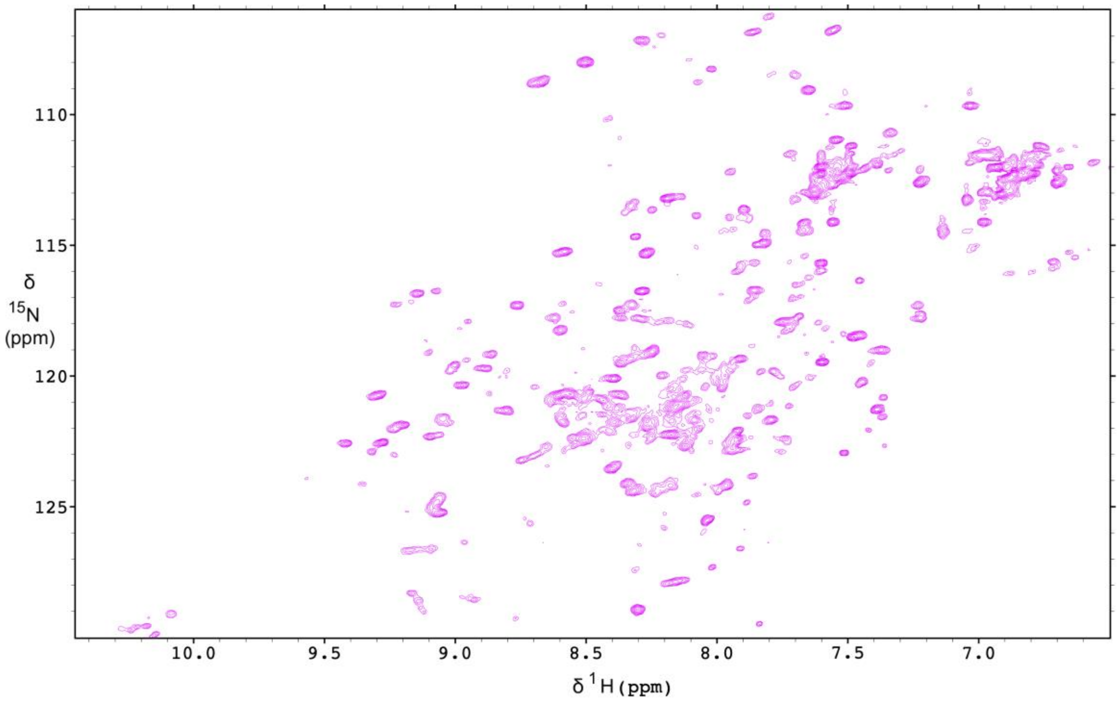

Supplement: Supplementary file 1 [file ijms-22-01439-s001.pdf]
